# Immunomodulatory effects of HUC-MSCs therapy: enhancing adaptive and innate immune responses in aging frailty

**DOI:** 10.3389/fimmu.2026.1796161

**Published:** 2026-07-13

**Authors:** Ce Huang, Yingqian Zhu, Xue Gong, Shengyu Feng, Guangying Huo, Hailiang Liu, Hua Jiang, Zhongmin Liu

**Affiliations:** 1 Institute for Regenerative Medicine, State Key Laboratory of Cardiology and Medical Innovation Center, Shanghai East Hospital, School of Medicine, Tongji University, Shanghai, China; 2 Institutes of Biomedical Sciences, Shanghai Institute of Infectious Disease and Biosecurity, Fudan University, Shanghai, China; 3 Department of Geriatrics, Shanghai East Hospital, Tongji University School of Medicine, Shanghai, China; 4 Department of General Medicine, Shanghai East Hospital, Tongji University School of Medicine, Shanghai, China; 5 Key Laboratory of Xinjiang Phytomedicine Resource and Utilization of Ministry of Education, College of Life Sciences, Shihezi University, Shihezi, China; 6 Institute of Advanced Biotechnology and School of Medicine, Southern University of Science and Technology, Shenzhen, China

**Keywords:** human umbilical cord-derived mesenchymal stem cells (HUC-MSCs), aging frailty, single-cell RNA sequencing, mucosal-associated invariant T (MAIT) cells, cell-cell communication, immunosenescence

## Abstract

**Background:**

Frailty associated with aging, characterized by chronic inflammation and immune dysfunction, poses a significant public health challenge. Human umbilical cord-derived mesenchymal stem cells (HUC-MSCs) exhibit immunomodulatory properties; however, their mechanism of action in frail elderly populations remains unclear. Building on a prior clinical trial investigating HUC-MSCs for elderly frailty (Trial Registration: ClinicalTrials.gov, NCT04314011; PubMed ID: 38679727), this study was conducted to further elucidate the mechanism of action of HUC-MSCs.

**Methods:**

We performed a longitudinal single-cell RNA sequencing (scRNA-seq) study on peripheral blood mononuclear cells (PBMCs) from frail elderly patients before and after HUC-MSCs therapy. Immune cell dynamics were analyzed across multiple time points, and cell-cell communication networks were reconstructed. Spearman correlation analyses were performed to link immune changes with clinical frailty and physical performance metrics.

**Results:**

HUC-MSCs induced a time-dependent recalibration of the immune system. An early, transient expansion of MAIT cells (p=0.049) was followed by a sustained reduction in B cell hyperactivity (p=0.032) and a phenotypic shift in naïve B cells. NK cell cytotoxicity was enhanced (increased GNLY), while NKT cells showed modulated stress-response signatures. Cell-cell communication was reorganized, with the EPHA/NRG pathways highlighting CD4^+^ TEM, MAIT, and NKT cells as central signaling hubs. Critically, these immunomodulatory changes were quantitatively associated with clinical improvement: reductions in B cell and innate immune subsets correlated with gains in grip strength and gait speed, while regulatory T cell expansion was linked to improved lower-limb function.

**Conclusions:**

HUC-MSCs restore immune homeostasis in aging frailty through sequential, multi-faceted immunomodulation. The association between specific immune remodeling features and functional recovery positions HUC-MSCs as a promising mechanism-based therapy for aging frailty.

## Graphical Abstract

**Figure:**
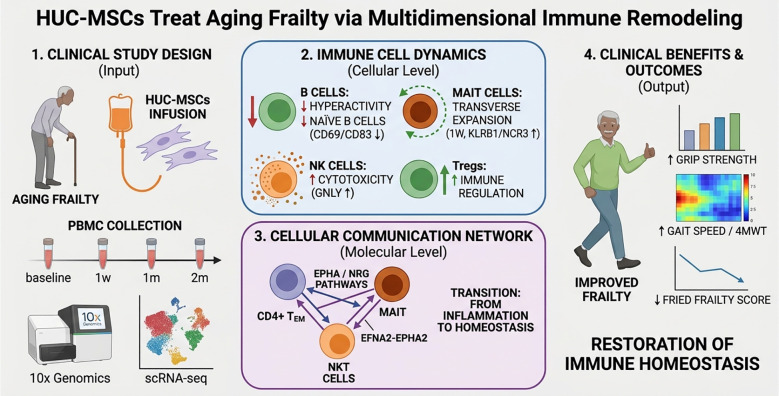

## 1 Introduction

Aging frailty is a complex geriatric syndrome characterized by declines in physiological reserves across multiple organ systems and diminished capacity to maintain homeostasis (1, 2). Frail older individuals exhibit increased vulnerability to adverse outcomes, including disability, hospitalization, and mortality (3, 4). Growing evidence suggests that inflammageing and immunosenescence are the central biological mechanisms underlying frailty (5–7). The immunosenescence, a hallmark of aging, characterized by immune alterations include disrupted naïve/memory ratio in T and B cells, inadequate antigen response, expansion of senescent immune cells, and impaired adaptive immunity (8, 9), all of which play key roles to the diminished host resilience. Importantly, age-related changes in peripheral blood mononuclear cells (PBMCs) occur even in healthy older adults, raising the possibility that alterations in immune may reflect age-related or frailty-associated immune states. The PBMC profiles in healthy aging are characterized by a gradual decline in naïve T cell populations, contraction of T cell receptor (TCR) diversity, and expansion of memory T cell clonotypes (10). In contrast, emerging evidence suggests that frailty is associated with distinct immune alternations beyond those observed in healthy aging. Aging individuals with frailty exhibit divergent differentiation trajectories of naïve CD4^+^ T cells and a more restricted TCR. The study also observed the expansion of cytomegalovirus-associated CD8^+^ T cell clones and pro-inflammatory immune signatures, such as increased expression of chemokines such as CCL3 and CCL4 in innate immune cells, indicating the shift toward a more proinflammatory immune landscape in frailty (6).

Current management strategies for frailty primarily rely on non-pharmacological, multimodal interventions, including exercise, nutritional supplementation, and comprehensive geriatric care (11–13). However, the overall efficacy of these intervention remains modest with heterogeneous responses across individuals (14, 15).

Mesenchymal stem cells (MSCs), particularly human umbilical cord-derived mesenchymal stem cells (HUC-MSCs), have emerged as a promising candidate due to their immunomodulatory, anti-inflammatory, and regenerative properties (16, 17). Preclinical studies have demonstrated that MSCs can attenuate systemic inflammation, modulate immune responses, and promote tissue repair (18, 19), while clinical trials report improved physical performance in frail patients (20, 21). In our previous randomized, double-blind, placebo-controlled trial, the results have demonstrated that intravenous infusion of HUC-MSCs improved quality of life, physical performance with reduced pro-inflammatory cytokines in frail older adults, and the intravenous infusion of HUC-MSCs was safe (22). The findings suggest that MSC therapy may linked to its immunomodulatory effects, although the underlying cellular mechanisms remain to be fully elucidated.

Recent advances in single-cell RNA sequencing (scRNA-seq) enabled high-resolution profiling of immune cell heterogeneity and dynamic cellular responses to therapeutic interventions (23, 24). Here, we employed scRNA-seq to explore immunomodulatory effects of HUC-MSCs in aging frailty, aiming to characterize MSC-induced changes in adaptive and innate immune compartments, and identify key immune cell subsets associated with therapeutic response.

## 2 Materials and methods

### 2.1 Study Cohort

This study is a follow-up analysis based on a prior prospective clinical trial (22). The original trial was conducted at Shanghai East Hospital (Shanghai, China) and enrolled frail adults aged 60–80 years, with frailty diagnosed according to Fried’s phenotypic criteria (2). Eligible participants were required to have a life expectancy exceeding 12 months and no history of active infections, malignancies, or concurrent participation in other clinical trials. Key exclusion criteria comprised severe cardiovascular comorbidities (e.g., NYHA class III/IV heart failure, acute coronary syndrome within the past 6 months, or uncontrolled hypertension ≥180/110 mmHg), advanced organ dysfunction (eGFR <30 mL/min/1.73 m² or Child-Pugh C cirrhosis), metabolic dysregulation (HbA1c >9% or active autoimmune diseases), substance abuse, or cognitive impairment affecting compliance.

The present follow-up analysis focused on performing PBMCs obtained from 12 participants enrolled in the aforementioned trial. The study protocol remained consistent with the original trial: participants were randomly assigned under double-blind conditions to receive intravenous infusions. The intervention group received HUC-MSCs at a dose of 1 × 10^6^ cells/Kg, while the control group received a placebo (normal saline). Infusions were administered at baseline and again at the 1-month follow-up visit. Detailed demographic and clinical characteristics of the participants, including frailty scores and comorbidities, are provided in **Supplementary Table 1**. The overall physical performance which encompassed grip strength, four-meter walking test (4MWT), timed up and go (TUG) test, five times sit to stand test (FTSST), were evaluated at 1-month and 2-month post infusion to examine the ability to stand or movement (**Supplementary Table 2**).

### 2.2 HUC-MSCs Preparation

Clinical-grade HUC-MSCs were isolated from term-delivered umbilical cords of healthy donors under written informed consent. The HUC-MSCs were manufactured as previously described (25, 26). Tissue processing and expansion were conducted in a GMP-certified facility, adhering to Good Manufacturing Practice guidelines. Following meticulous removal of blood vessels, the umbilical cord tissues were dissected into 0.5 cm³ explants and cultured in α-MEM medium (Gibco) supplemented with 10% fetal bovine serum (FBS; Gibco) and 1% penicillin/streptomycin at 37 °C with 5% CO_2_. Cells harvested between passages 3–4 were used for therapeutic infusion. HUC-MSCs underwent rigorous characterization to ensure quality and safety. Surface marker profiling via flow cytometry confirmed expression of CD73, CD90, and CD105, while lacking CD34 and CD45. Trilineage differentiation potential was validated using osteogenic (STEMPRO Osteogenesis Kit), adipogenic (STEMPRO Adipogenesis Kit), and chondrogenic (STEMPRO Chondrogenesis Kit) induction media (Thermo Fisher). Safety assessments included sterility testing (bacteria, mycoplasma, fungi), viral screening (syphilis), and endotoxin quantification (≤0.5 EU/mL) via Limulus amebocyte lysate assay. These protocols ensured compliance with international cell therapy standards while maintaining functional potency for clinical application.

### 2.3 Sample collection and processing

Longitudinal peripheral blood samples (6 mL) were collected from fasting participants using EDTA anticoagulant tubes at four timepoints: baseline (pre-treatment), 1 week, 1 month, and 2 months post-initial hUC-MSC infusion. Within 2 hours of collection, blood was diluted 1:1 with sterile PBS (Gibco) and subjected to density gradient centrifugation (Ficoll-Paque PLUS, Cytiva) in 15 mL conical tubes at 400 × g for 30 min (room temperature, brake disabled). PBMCs were carefully aspirated from the plasma-Ficoll interface, washed thrice with PBS (300 × g, 10 min), and treated with ACK lysing buffer (BioLegend) for 5 min at 4 °C to remove residual erythrocytes. Viable cells (>95% by trypan blue exclusion) were cryopreserved in freezing medium containing 90% heat-inactivated FBS (Gibco) and 10% DMSO (Sigma-Aldrich), with controlled-rate freezing followed by long-term storage in liquid nitrogen vapor phase (-150 °C).

### 2.4 Library preparation for scRNA-seq

Single-cell RNA sequencing libraries were constructed using the Chromium Next GEM Single Cell 3’ Kit v3.1 (10 x Genomics, PN-1000268) following the manufacturer’s protocol. Briefly, single-cell suspensions with >85% viability (Trypan Blue exclusion) were loaded onto the Chromium Controller to generate Gel Bead-in-Emulsions (GEMs) at a target capture of 5,000 cells per sample. Reverse transcription was performed within GEMs using Maxima H Minus Reverse Transcriptase (Thermo Fisher) under the following thermal cycling conditions: 53 °C for 45 min, 85 °C for 5 min, followed by hold at 4 °C. Complementary DNA (cDNA) was purified using Dynabeads MyOne Silane Beads (10 x Genomics), amplified for 10 cycles (98 °C for 45 sec; 98 °C for 20 sec, 67 °C for 30 sec, 72 °C for 1 min ×10 cycles; 72 °C for 1 min), and enzymatically fragmented (37 °C, 5 min). Final libraries were quantified via Qubit 4.0 Fluorometer (Thermo Fisher) and assessed for size distribution using the Agilent 2100 Bioanalyzer High Sensitivity DNA Kit (Agilent, 5067-4626), demonstrating expected profiles (peak ~400 bp). Paired-end sequencing (150 bp ×2) was performed on the Illumina NovaSeq 6000 platform (Illumina) with a target depth of 50,000 reads per cell.

### 2.5 Preprocessing of scRNA-seq data and subsequent downstream analyses

Raw sequencing data were processed through the 10x Genomics Cell Ranger pipeline (v3.1.0) with default parameters to generate a cell-by-gene UMI count matrix. Subsequent analyses were performed using Seurat (v3.2.3) under R (v4.1.2), beginning with stringent quality control to exclude low-quality cells based on transcriptome complexity (200 < detected genes < 8,000), mitochondrial read contamination (≤15%), and ribosomal gene content (≤5%). Log-normalized data were scaled to regress out confounding factors (mitochondrial percentage, sequencing depth) prior to dimensionality reduction *via* principal component analysis (PCA), where the top 50 principal components identified by ElbowPlot were selected for UMAP projection (min.dist=0.3, spread=1.0). To correct for potential technical batch effects across samples, we applied Harmony (27) on the top 50 principal components, using sample identity (orig.ident) as the batch variable. The Harmony-corrected embeddings were then used for all downstream uniform manifold approximation and projection (UMAP) visualization and clustering (**Supplementary Figure 1**). Cell clusters were resolved through shared nearest neighbor (SNN) clustering at a resolution of 0.5, with population identities annotated using a multi-modal approach: (1) marker gene identification *via* Wilcoxon rank-sum test (FindAllMarkers, logfc.threshold =0.25); (2) reference mapping against the Human Primary Cell Atlas using SingleR (v1.8.1); (3) validation through CellMarker 2.0 database (http://bio-bigdata.hrbmu.edu.cn/CellMarker/). For subgroup comparisons within specific cell types, differential expression analysis was performed using a dual strategy. First, to ensure the patient served as the unit of inference, we conducted a pseudobulk analysis by aggregating gene counts per patient per cell type, followed by differential testing using DESeq2. Second, as an exploratory approach to identify potential signals in this small cohort, we also performed cell-level differential expression analysis using the FindMarkersfunction in Seurat (Wilcoxon rank-sum test) with stringent multi-layered criteria: a log2 fold-change threshold > 0.25, Bonferroni-adjusted *p* < 0.05, and requirement for gene detection in a minimum percentage of cells. Results from both approaches are reported, with the pseudobulk analysis considered the more conservative benchmark, followed by functional enrichment analysis via clusterProfiler (v4.2.2) for Gene Ontology Biological Process terms. Visualization of UMAP embeddings and gene expression patterns was achieved using ggplot2 (v3.3.5), the latter enabling kernel density estimation for spatial expression mapping.

### 2.6 Ligand-receptor interaction analysis

Ligand-receptor interaction analysis was performed using the R package CellChat (v1.6.0) to systematically decode intercellular communication dynamics across experimental conditions. Single-cell transcriptomic data were integrated with the CellChatDB ligand-receptor interaction database (v1.0), which curates 2,021 validated human signaling pairs across 13 major pathways (e.g., Wnt, TGF-β, chemokine). Cell-cell communication networks were constructed by calculating interaction probabilities through a mass action law-based model, incorporating both expression specificity of ligand-receptor pairs and spatial proximity weights derived from cell type colocalization patterns. Significant interactions were identified using permutation testing (1,000 iterations, p<0.05) with Benjamini-Hochberg correction for multiple comparisons. Communication strength was quantified via the information flow metric, defined as the sum of interaction probabilities between sender and receiver cell clusters. Differential network analysis between HUC-MSCs-treated and control groups was conducted using a Wilcoxon rank-sum test (p<0.01), revealing pathway-specific alterations in signaling intensity and topology. All analyses were stratified by timepoint (baseline, 1W, 1M, 2M) to capture temporal evolution of communication networks.

### 2.7 Correlation analysis between immune cell dynamics and clinical outcomes

To investigate the link between immune cell remodeling and clinical outcomes, we conducted Spearman rank correlation analyses. Patient-level changes in immune cell proportions (Δ = post-treatment − baseline) were correlated with changes in key clinical metrics—including the Fried frailty score, SPPB, TUG, 4MWT, grip strength, and FTSST—at 1 and 2 months post-infusion. For clinical metrics, Δ values were oriented so that a positive value indicates improvement (e.g., lower scores for Fried, TUG, 4MWT, FTSST; higher scores for SPPB and grip strength). We also correlated changes in the mean expression of selected key genes (e.g., TUBB1 in megakaryocytes) with clinical changes. Due to the exploratory nature of the study and modest sample size, results are reported as raw p-values alongside Spearman’s ρ and sample sizes. A Benjamini–Hochberg correction was applied across all 204 tests performed; adjusted p-values are provided in **Supplementary Table 3**.

### 2.8 Statistical Analysis

Baseline characteristics between the two groups were compared using the independent-sample t-test or Mann–Whitney U test based on the normality of data distribution. Within-group changes across baseline, 1-month and 2-month follow-up were evaluated by the paired t-test or Wilcoxon signed-rank test accordingly. For multiple pairwise comparisons among the three time points, the Bonferroni correction was applied, and the corrected significance level was set at α = 0.05/3 ≈ 0.017. Categorical variables were presented as n/N (%) and analyzed using the chi-square test. For all analyses comparing immune cell type proportions between the HUC-MSC and control groups, the unit of analysis was the patient. The proportion of each cell type was calculated per patient per time point. Group comparisons at each time point were then performed using the Mann-Whitney U test on these patient-level values (n=7 HUC-MSC, n=5 control). Longitudinal trends and group×time interaction effects over different time points were assessed using linear mixed-effects models with maximum likelihood estimation. A P value < 0.05 was considered statistically significant for overall main effects, while pairwise comparisons adopted the Bonferroni-corrected threshold of P < 0.017.

## 3 Results

### 3.1 scRNA-seq Reveals Dynamic Immune Cell Remodeling Post-HUC-MSCs Therapy

At baseline, no significant differences were observed between the two groups. Following intervention, the MSC-treated group showed improvements in frailty status and physical function compared with the control group, including reductions in Fried frailty scores and improvements in mobility-related measures (e.g., TUG and 4MWT) and FTSST. The findings of changes of Fried frailty phenotype scales and overall physical performance were presented in **Supplementary Table 2**.

**Figure 1:**
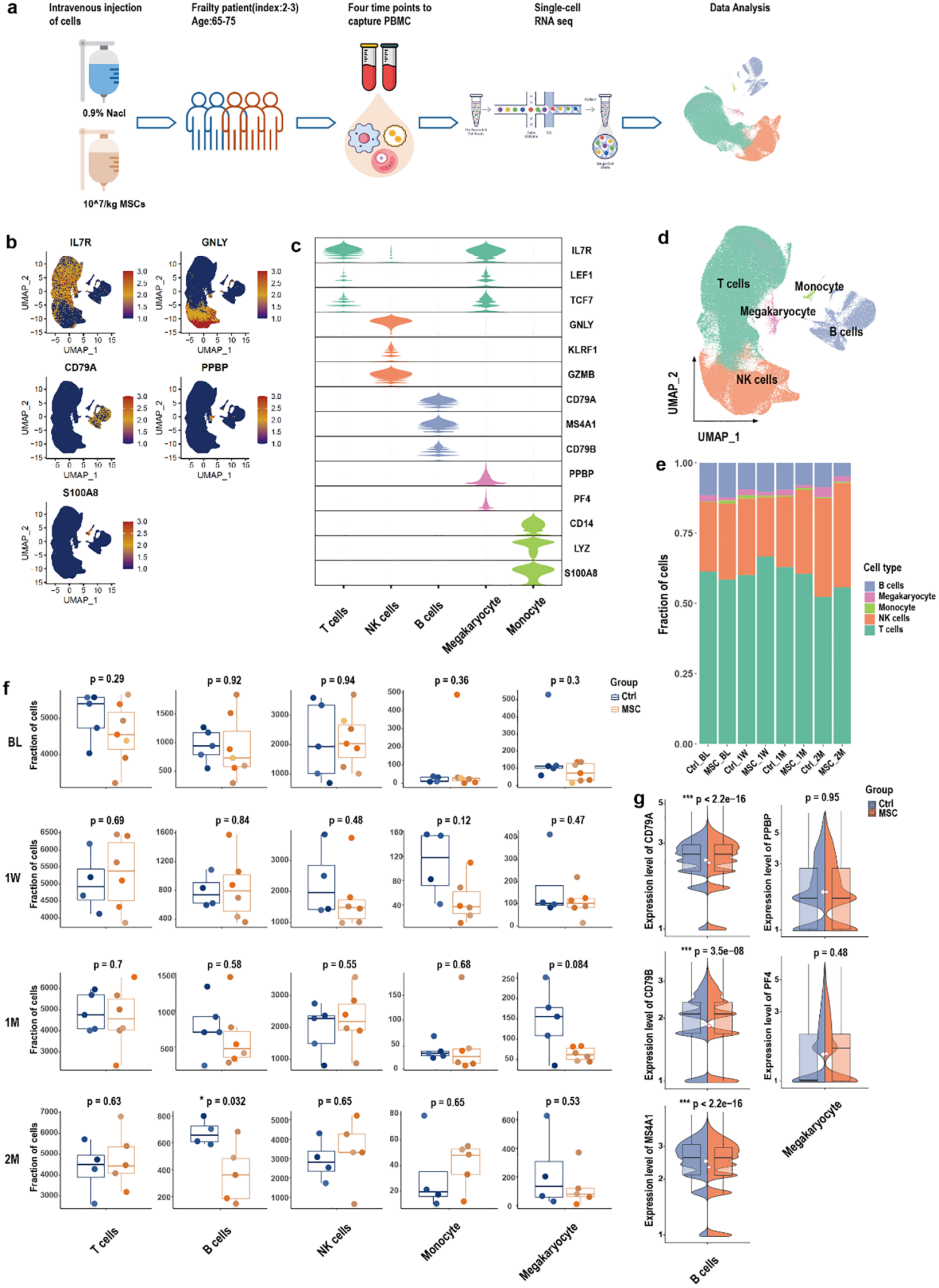
Single-cell transcriptomic profiling reveals HUC-MSCs-mediated immune modulation in aging frailty. **(a)** Study design: Twelve frail elderly patients were randomized into HUC-MSCs-treated (*n* = 7) and placebo groups (*n* = 5), with blood sampling at BL, 1W, 1M, and 2M. **(b)** Heatmap of lineage-specific marker gene expression (*IL7R* [T cells], *GNLY* [NK cells], *CD79A* [B cells], *CD14* [monocytes], *PPBP* [megakaryocytes]). **(c)** Violin plots quantify canonical marker expression across cell types. **(d)** UMAP visualization of 339,437 PBMCs clustered into five major immune lineages. **(e)** Horizontal bar plots depict cell type proportions. **(f)** Dynamic changes in immune subset distribution between HUC-MSCs and placebo groups over time. HUC-MSCs treatment significantly reduced B cell proportions at 2M (*p*=*0.032*) and megakaryocyte abundance at 1M (*p=0.08*). **(g)** Downregulation of B cell markers (*CD79A*, *MS4A1*; *p<0.0001)* in the HUC-MSCs group. Statistical significance was determined by Mann-Whitney U test (*p<0.05, ***p<0.0001).

Overall, these findings indicate that MSC therapy is associated with enhanced functional performance in frail older adults. Then, we performed scRNA-seq on PBMCs from 12 individuals with age-related frailty to investigate the immunomodulatory potential of HUC-MSCs therapy in frail elderly patients. Participants were randomized into HUC-MSCs-treated (*n* = 7) and placebo groups (*n* = 5), with matched age, sex, and ethnicity (**Figure 1a**; **Supplementary Table 1**). Blood samples (*n* = 42) were collected at baseline (BL), 1 week (1W), 1 month (1M), and 2 months (2M) post-intervention. Sequencing libraries generated via the 10x Genomics platform were processed using Cell Ranger (v3.1.0), yielding a high-quality transcriptomic dataset of 339,437 cells after stringent quality control. Unsupervised clustering and UMAP visualization identified five major immune lineages: T cells (markers: *IL7R*, *LEF1*), NK cells (*GNLY*, *GZMB*), B cells (*CD79A*, *MS4A1*), monocytes (*CD14*, *LYZ*), and megakaryocytes (*PPBP*, *PF4*) (**Figures 1b-e**; **Supplementary Table 4**). Comparative analysis revealed distinct immunomodulatory effects of HUC-MSCs therapy. At 2 months, the treated group exhibited a significant reduction in B cell proportions compared to placebo (*p* = 0.032; **Figure 1f**), accompanied by downregulation of B cell-specific markers (*CD79A*, *MS4A1*; *p* < 0.0001; **Figure 1g**). Megakaryocyte abundance also trended lower in the HUC-MSCs group at the final follow-up, though inter-group differences across other clusters were non-significant (Mann-Whitney U test, *p*>0.05; **Figure 1f**). These findings highlight HUC-MSCs-driven alterations in specific immune subsets, particularly B cells, suggesting targeted modulation of adaptive immunity in aging frailty.

### 3.2 HUC-MSCs Therapy Reprograms B Cell Phenotypes in Aging Frailty

**Figure 2:**
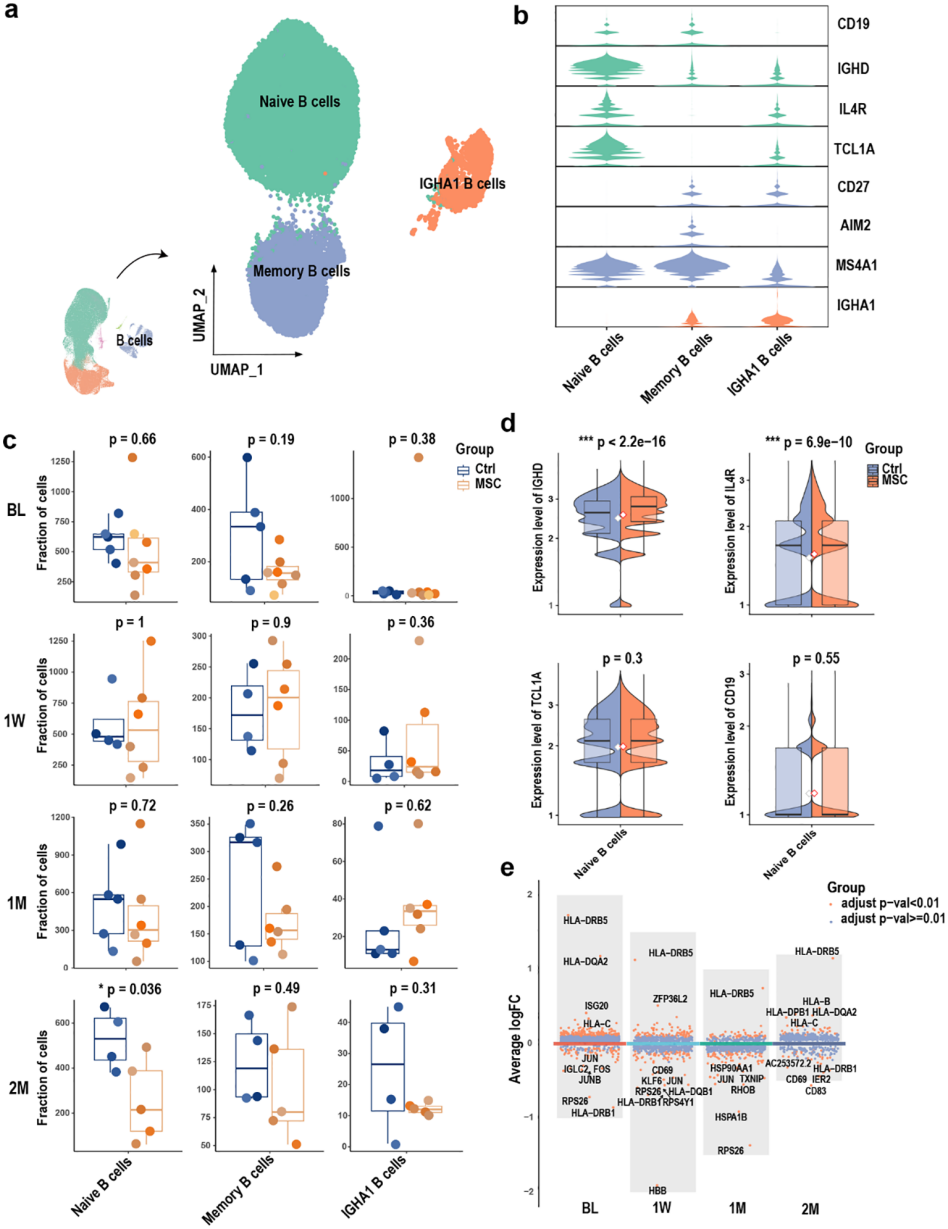
HUC-MSCs therapy reprograms B cell heterogeneity and gene expression in aging frailty. **(a)** UMAP clustering of B cells (n=31,591 cells), color-coded by subtype: naïve (green), memory (blue), and IGHA1^+^ B cells (orange). **(b)** Violin plots quantify signature gene expression (*IGHD* [naïve], *CD27* [memory], *IGHA1* [mucosal]) across subsets. **(c)** Temporal shifts in B cell subtype proportions: HUC-MSCs-treated group exhibited reduced naïve B cells at 2 months (*p* = 0.036) versus placebo. **(d)** Upregulation of naïve B cell markers (*IGHD*, *IL4R*; *p* < 0.0001) in the HUC-MSCs group. **(e)** Volcano plot highlights downregulated activation markers (*CD69*, *CD83*) and stress-response genes (*TXNIP*, *HSPA1B*) post-treatment. Statistical significance was determined by Mann-Whitney U test (*p < 0.05, ***p < 0.0001).

To dissect the immunophenotypic remodeling of B cells induced by HUC-MSCs therapy, we performed UMAP-based clustering on 339,437 PBMCs, identifying three distinct B cell subsets: naïve B cells (*CD19*^+^*IGHD*^+^*IL4R*^+^), memory B cells (*CD27*^+^*AIM2*^+^), and IgA-producing IGHA1^+^ B cells (**Figures 2a, b**). Longitudinal analysis revealed a significant reduction in naïve B cell frequency in the HUC-MSCs group at 2 months post-treatment compared to placebo (*p* = 0.036; **Figure 2c**), accompanied by upregulated *IGHD* and *IL4R* expression (*p* < 0.0001; **Figure 2d**). Notably, this naïve B cell decline correlated with suppressed activation markers (*CD69*, *CD83*; **Figure 2e**) and downregulated oxidative stress-associated genes (*TXNIP*, *HSPA1B*; **Figure 2e**), suggesting HUC-MSCs attenuate B cell activation while promoting an anti-inflammatory state. Although no concomitant increase in memory B cells was observed, the sustained reduction of naïve B cells implies a potential shift toward antigen-experienced subsets, a phenomenon previously linked to MSC-mediated immune tolerance. These findings collectively demonstrate that HUC-MSCs reshape the B cell landscape by modulating both subset distribution and functional states, which may contribute to restoring immune equilibrium in aging-related frailty.

### 3.3 HUC-MSCs Therapy Modulates Innate Immunity and Megakaryocyte Dynamics in Aging Frailty

**Figure 3:**
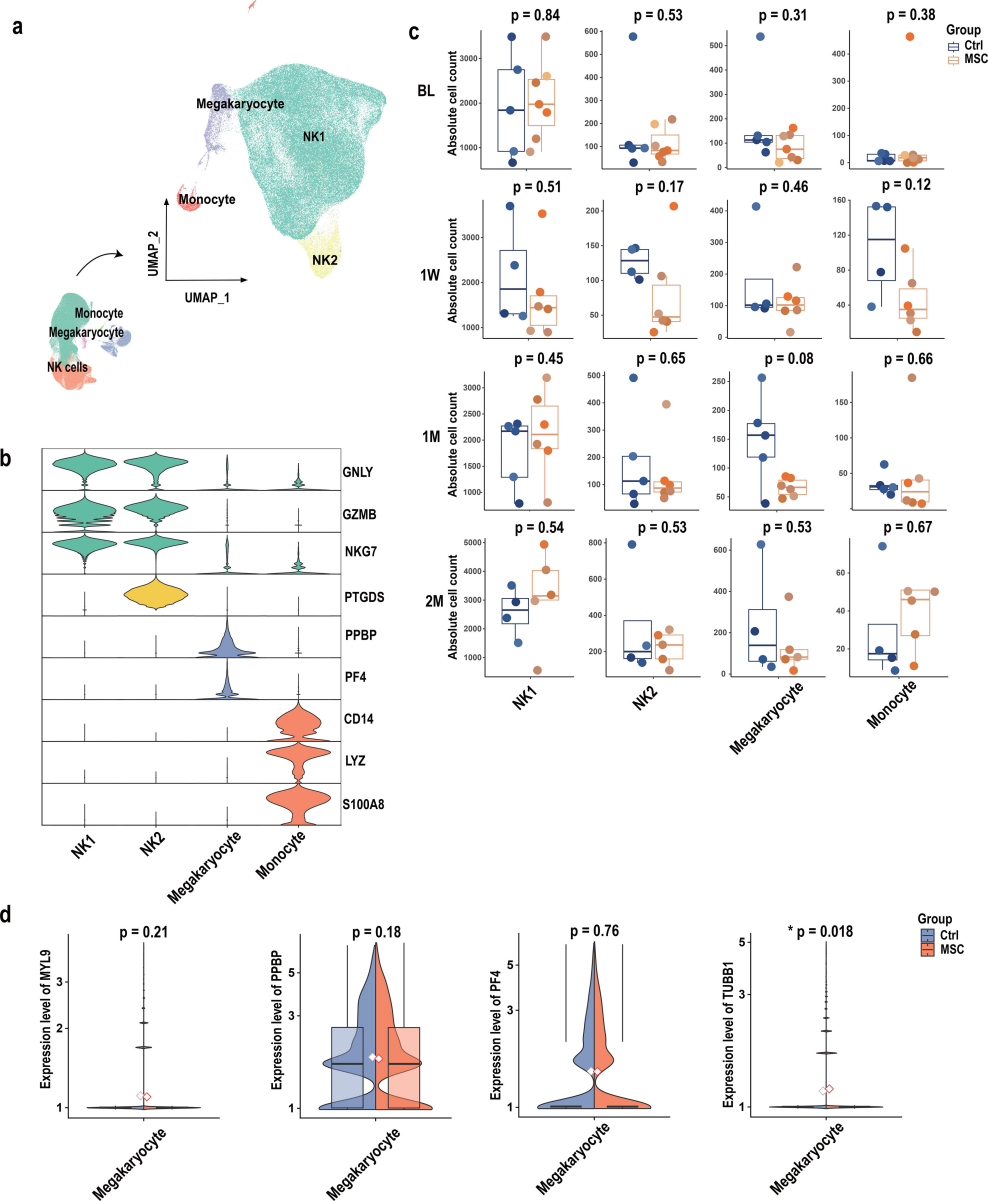
HUC-MSCs therapy modulates innate immune and megakaryocyte transcriptional profiles in aging frailty. **(a)** UMAP clustering of 104,916 innate immune cells (NK1: *GNLY*^+^, NK2: *PTGDS*^+^) and megakaryocytes (*PPBP*^+^), color-coded by subtype. **(b)** Violin plots quantify signature gene expression (*GNLY* [cytotoxicity], *CD14* [monocytes], *PPBP* [megakaryopoiesis]). **(c)** Temporal dynamics of cell subtype proportions: HUC-MSCs-treated group showed reduced megakaryocytes at 1M (*p* = 0.08) versus placebo. **(d)** Upregulation of megakaryocyte maturation marker *TUBB1* (*p* = 0.018) in the HUC-MSCs group. **(e)** A volcano plot is utilized to illustrate the differentially expressed genes (DEGs). Statistical significance: Mann-Whitney U test (*p < 0.05).

To delineate the impact of HUC-MSCs therapy on innate immunity and megakaryocyte dynamics in aging frailty, we analyzed 339,437 PBMCs, focusing on NK cell subsets (NK1: *GNLY^+^GZMB^+^*; NK2: *PTGDS*^+^), monocytes (*CD14*^+^*S100A8*^+^), and megakaryocytes (*PPBP*^+^*PF4*^+^, markers of platelet degranulation (28)) (**Figures 3a, b**). The HUC-MSCs group exhibited a trend toward reduced megakaryocyte abundance at 1 month (*p* = 0.08), accompanied by upregulated *TUBB1* (a β-tubulin isoform critical for cytoskeletal integrity) expression (*p* = 0.018), suggesting cytoskeletal reorganization (**Figures 3c, d**). Single-cell analysis further identified distinct transcriptional changes within the *PPBP*^+^*PF4*^+^ megakaryocyte population, which may be associated with functional modulation. NK1/NK2 subsets and monocytes maintained stable proportions post-treatment (p>0.05), while transcriptomic profiling revealed nuanced functional alterations, such as *PTGDS*^+^ NK2 cells associated with lipid mediator synthesis. Concurrently, increased TUBB1 suggests enhanced megakaryocyte maturation, possibly optimizing platelet production while attenuating inflammatory crosstalk.

These findings highlight the dual role of HUC-MSCs therapy in modulating innate immunity and megakaryocyte biology. However, the functional relevance of the observed transcriptional changes, including *TUBB1* upregulation, requires validation through mechanistic studies (e.g., CRISPR knockdown) and larger clinical cohorts to assess long-term implications for platelet function and inflammaging.

### 3.4 HUC-MSCs Therapy Recalibrates Adaptive T Cell Immunity to Restore Immune Equilibrium in Aging Frailty

**Figure 4:**
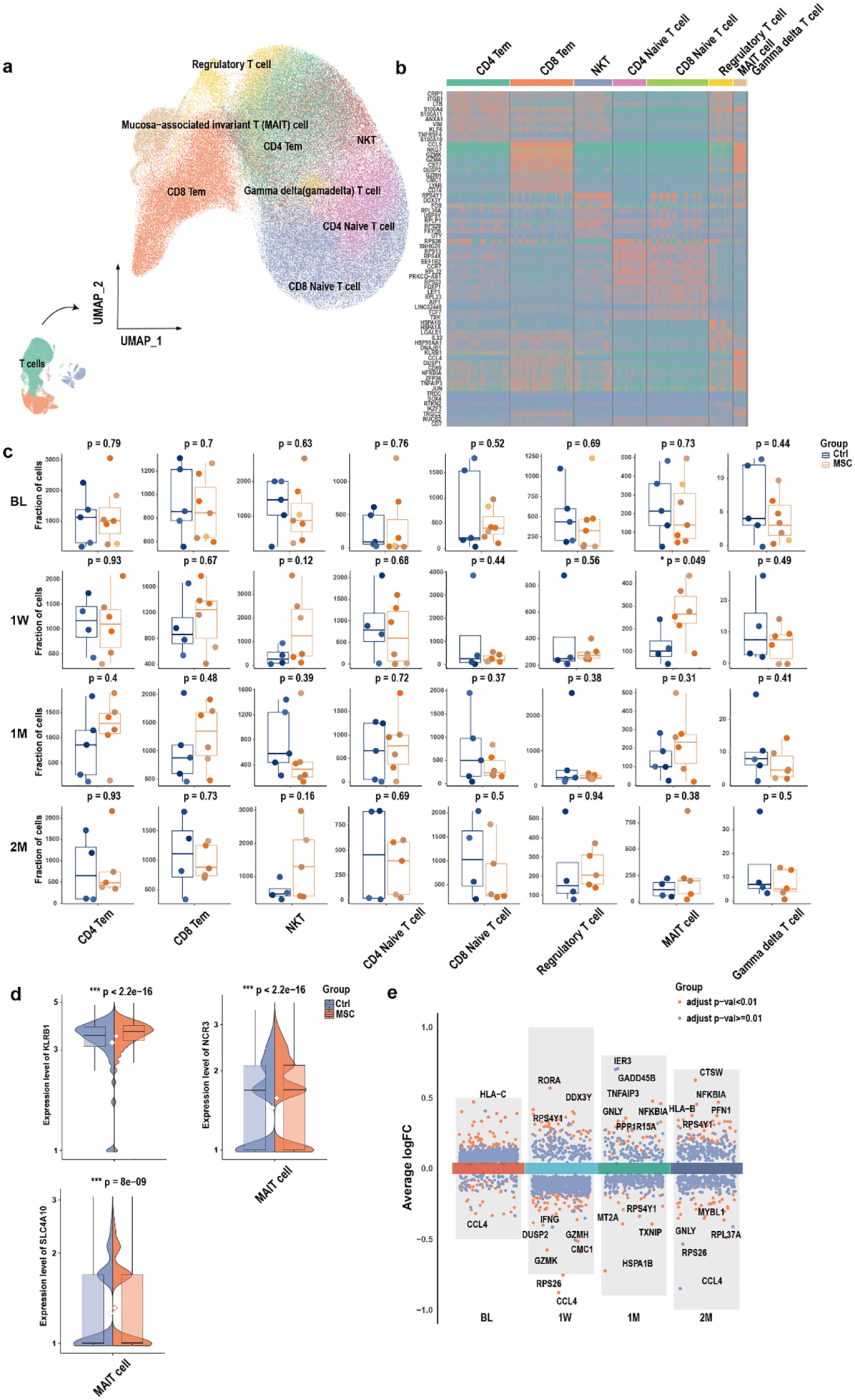
HUC-MSCs therapy drives dynamic T cell remodeling in aging frailty. **(a)** UMAP clustering of 202,930 T cells, color-coded by subtype: CD4^+^ naïve, CD8^+^ effector memory, MAIT, and regulatory T cells. **(b)** Heatmap of scaled expression for subset-defining genes (*CRIP1* [CD4^+^ TEM], *CCL5* [CD8^+^ TEM], *KLRB1* [MAIT]). **(c)** Temporal shifts in T cell subset proportions: MAIT cells transiently expanded at 1W in the HUC-MSCs group (*p* = 0.049) versus placebo. **(d)** Upregulation of MAIT markers (*KLRB1*, *SLC4A10*, *NCR3*; *p<0.0001*) post-treatment. **(e)** The volcano plot illustrates the differential expression of genes across four specific time points. Statistical significance: Mann-Whitney U test (*p<0.05, ***p<0.0001).

To elucidate the impact of HUC-MSCs therapy on adaptive T cell immunity in aging-related frailty, we re-clustered the T cell landscape *via* UMAP analysis, identifying eight distinct subpopulations: CD4^+^ naïve T cells, CD8^+^ naïve T cells, CD8^+^ effector memory T cells (CD8^+^ TEM), CD4^+^ TEM, natural killer T (NKT) cells, mucosa-associated invariant T (MAIT) cells, gamma delta T cells, and regulatory T cells (**Figures 4a, b**). Differential gene expression analysis (**Figure 4e**; **Supplementary Table 5**) revealed proliferative signatures in CD4^+^ TEM and naïve subsets (*CRIP1*^+^, *S100A4*^+^), while CD8^+^ TEM exhibited cytotoxic potential marked by *CCL5* and *NKG7* overexpression. NKT cells showed enrichment in cytokine-related genes (*RPS4Y1*, *RPL36*), and MAIT cells demonstrated elevated activation markers (*CCL4*^+^, *CD69*^+^), consistent with their role in microbial metabolite recognition *via* MR1 (29).

Strikingly, MAIT cell frequency in peripheral blood surged at 1-week post-HUC-MSC treatment (*p* = 0.049; **Figure 4c**), accompanied by upregulated markers *KLRB1*, *NCR3*, and *SLC4A10* (*p* < 0.0001; **Figure 4d**). This transient expansion was followed by a gradual return to baseline levels at 1–2 months, suggesting a dynamic response. Based on the established biology of MAIT cells, this early expansion could represent a phase of enhanced systemic immune surveillance, while their subsequent decline might coincide with a transition towards tissue homing and inflammation resolution—processes often associated with factors like *IL-10* and *TGF-β* (30, 31). It is important to note that these specific functional roles (e.g., in pathogen control or senescent cell clearance) are proposed hypotheses based on prior literature and were not directly measured in this study. These exploratory findings invite the hypothesis that HUC-MSCs may transiently engage MAIT cells as part of a broader immunomodulatory sequence, potentially relevant for mitigating age-related immune dysfunction, a premise that requires direct functional validation.

### 3.5 HUC-MSCs Orchestrate Cell Type-Specific Transcriptional Remodeling to Restore Immune Homeostasis

**Figure 5:**
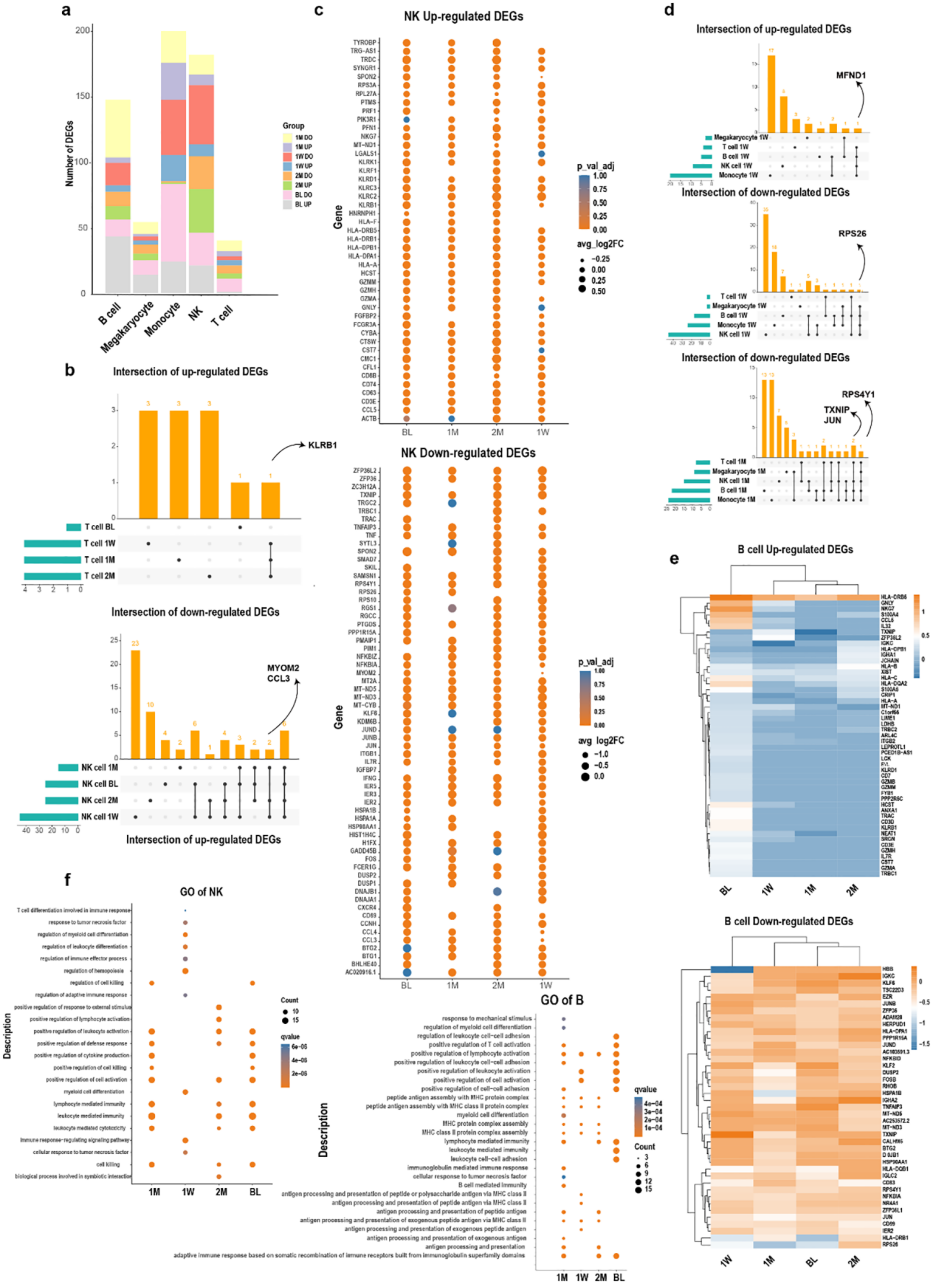
HUC-MSCs therapy induces coordinated transcriptional reprogramming across immune subsets. **(a)** Bar plot depicting the number of differentially expressed genes (DEGs) in major immune cell types at each post-treatment time point. A comparative increase in DEGs was observed in NK cells at 1 week. **(b)** Upset plot shows that *KLRB1* was commonly upregulated in T cells, while *MYOM2* and *CCL3* were commonly downregulated in NK cells across the three time points. **(c)** Dot plots highlighting the expression dynamics of selected DEGs in NK cell subsets. **(d)** Network diagram illustrating 23 genes that were coregulated and upregulated in T cells, B cells, and monocytes at 1 week, and 17 genes commonly downregulated in these subsets at 1 week and 1 month. **(e)** Heatmap depicting the expression patterns of DEGs in B cells, with color intensity representing normalized expression levels (blue: lower; orange: higher) across conditions. **(f)** Dot plots showing significantly enriched Gene Ontology (GO) terms associated with the DEG profiles of B cells and NK cells. Dot size represents the enrichment level, and color indicates the statistical significance of the association.

To comprehensively assess the transcriptional reprogramming associated with HUC-MSCs therapy, we analyzed differentially expressed genes (DEGs) across immune cell subsets at each post-treatment time point (adjusted *p* < 0.05). T cells exhibited relatively fewer transcriptional changes compared to B and NK cells (**Figure 5a** and **Supplementary Figure 2a**; **Supplementary Table 2**), with sustained upregulation of *KLRB1*—which encodes an inhibitory receptor—across all time points (**Figure 5b**). This persistent *KLRB1*expression suggests that HUC-MSCs may help modulate T cell activity, potentially contributing to a more regulated immune state. In contrast, NK cells showed a more dynamic transcriptional response, with the greatest number of DEGs observed at one-week post-infusion (**Figure 5a**). Notably, genes such as *MYOM2* and *CCL3* displayed consistent expression patterns throughout the study period (**Figure 5b**). Functional enrichment analysis revealed enhanced cytotoxic capabilities, including increased cell killing and cytokine production (**Figure 5f**), attributed to the upregulation of *GNLY* (**Figure 5c**). Simultaneously, the downregulation of pro-inflammatory genes (*DUSP2*, *HSPA1A*; **Figure 5c**) underscores the dual role of HUC-MSCs in enhancing innate immune surveillance while moderating inflammatory responses. B cells demonstrated a distinct pattern of activation and modulation, characterized by the downregulation of antigen presentation genes (*HLA-DRB5*, *HLA-DQA2*, *HLA-C*, and *NKG7*) and the upregulation of activation markers (*HLA-DRB1*, *RPS26*, *JUND*, *IGLC2*, and *EZR*) (**Figure 5e**). These alterations were associated with enriched pathways related to MHC assembly and T cell activation (**Figure 5f**), suggesting a modulated interaction between B and T cells. Naive B (NB) cells exhibited diminished activation, as evidenced by the downregulation of *CD69* (**Supplementary Figure S2b**), consistent with their immunoregulatory function *via* cytokine production pathways (**Supplementary Figure S2d**). MAIT cells displayed an anti-inflammatory reprogramming signature, characterized by the upregulation of *TNFAIP3* (**Supplementary Figure S2b**) and an enrichment in pathways associated with the negative regulation of inflammation (**Supplementary Figure S2c**). Similarly, NKT cells demonstrated a shift in stress-response signaling, marked by the upregulation of *PPDPF*, alongside the downregulation of *RPS11*, *FAM118A*, and *ITGB2* (**Supplementary Figure S2b**).

Collectively, HUC-MSCs orchestrate a multi-compartmental transcriptional reset: enhancing NK cell cytotoxicity, optimizing B cell antigen presentation, and fine-tuning MAIT/NKT cell inflammatory balance—a synergistic strategy to counteract immune dysregulation in aging frailty.

### 3.6 HUC-MSCs Reshape Intercellular Communication *via* EPHA/NRG Signaling to Enhance Immune Coordination

**Figure 6:**
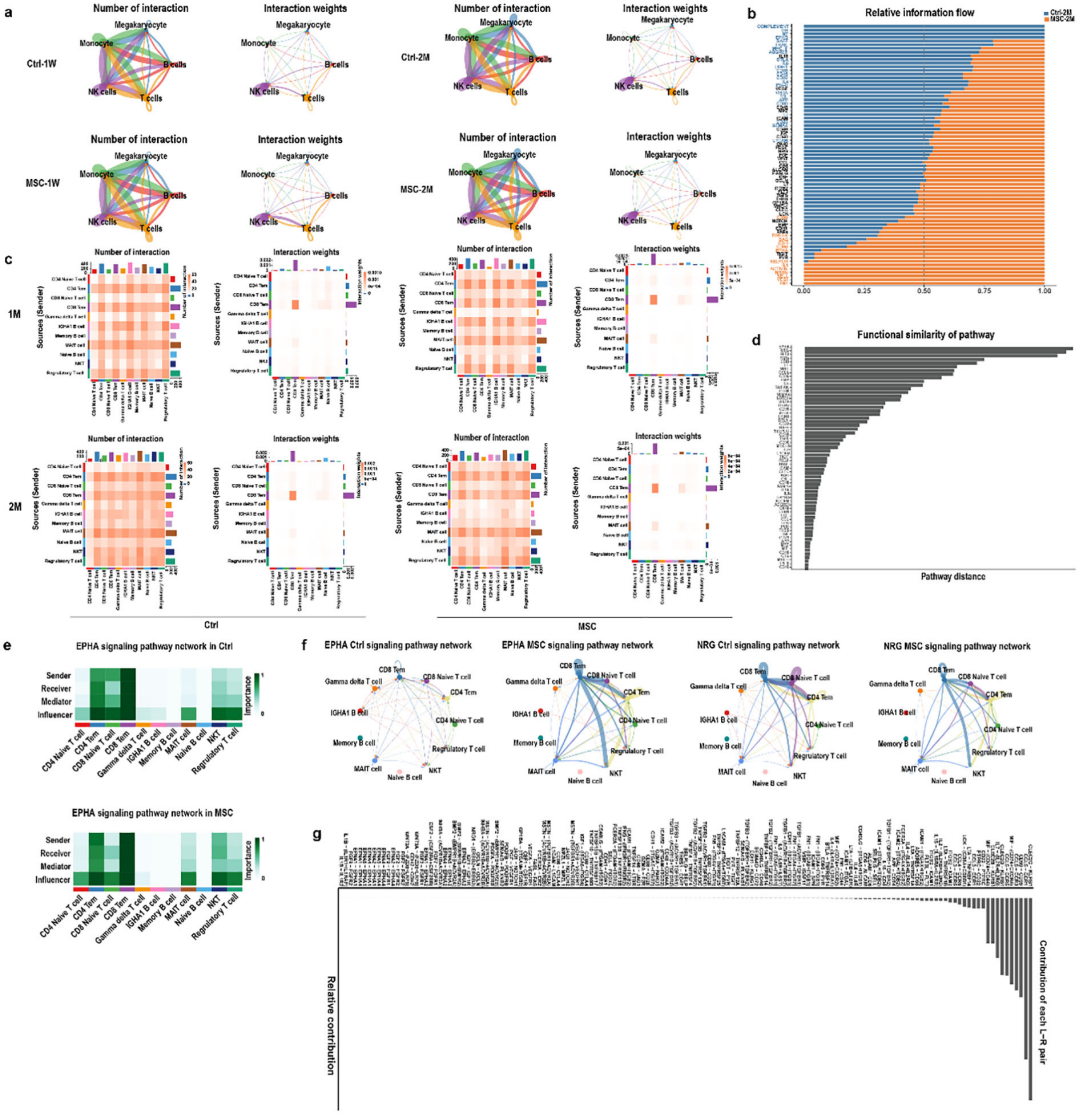
HUC-MSCs therapy reshapes intercellular communication networks through EPHA and NRG signaling pathways. **(a)** Global interaction networks showed stronger T, B, and NK cell interactions at 1 week and 2 months post-treatment compared to baseline and 1 month. **(b)** T-B cell crosstalk: HUC-MSC-treated group exhibited enhanced communication strength between CD8^+^TEM and MAIT cells. **(c)** Pathway-specific information flow: EPHA and NRG signaling were significantly amplified in the HUC-MSC group. **(d)** Pathway divergence: EPHA signaling showed the largest functional dissimilarity between groups, driven by shifted sender-receiver patterns. **(e)** EPHA network topology: CD4^+^ TEM and MAIT cells became dominant signal senders in the HUC-MSC group (node size: centrality score). **(f)** Enhanced EPHA and NRG signaling between CD8^+^ TEM and NKT cells. **(g)** EFNA2-EPHA2 ligand-receptor pair emerged as the most significant contributor to the EPHA signaling network in the group treated with MSCs, implicating its pivotal role in immune coordination.

To investigate how HUC-MSCs therapy reprograms immune communication in aging frailty, we integrated single-cell transcriptomic profiling with CellChat’s ligand-receptor interaction analysis. Longitudinal tracking revealed dynamic network remodeling, with T, B, and NK cells showing progressively strengthened interactions at 1-week and 2-month post-treatment intervals compared to stable baseline and 1-month profiles (**Figure 6a** and **Supplementary Figure S3a**). Notably, CD8^+^ TEM and MAIT cells exhibited time-dependent enhancement of communication signals, reaching peak intensity at the 2-month follow-up, highlighting MSC-driven optimization of adaptive immune coordination (**Figure 6b** and **Supplementary Figure S3b**).

HUC-MSC-treated groups activated tissue-repair pathways (FN1, VEGI, SELL, MSTN, SELPLG, EPHA), while control groups-maintained inflammation-associated signaling (GH, NT, MHC-II, IL16) (**Figure 6c**). The EPHA pathway demonstrated the most significant restructuring (**Figure 6d**), characterized by reduced signaling initiation from CD8 Naïve T cells, amplified sender activity in CD4 Tem/MAIT/NKT cells, and polarized receiver roles favoring MAIT/NKT populations over CD4 Tem cells (**Figure 6e**). A parallel shift occurred in the NRG pathway, where MSC treatment redirected signaling dominance to CD4 Tem/MAIT/NKT clusters (**Supplementary Figure S3c**). Network visualization confirmed enhanced EPHA signaling across adaptive and innate lymphocyte subsets, alongside amplified NRG activity in cytotoxic cell populations (**Figure 6f**). Central to this reorganization was the EFNA2-EPHA2 ligand-receptor pair, which emerged as the primary driver of EPHA pathway activation, underscoring ephrin-mediated cellular remodeling as a key mechanism (**Figure 6g**).

This reprogramming strategy operates through dual-phase modulation: boosting proactive communication *via* CD4^+^ TEM/MAIT/NKT-driven EPHA/NRG networks while suppressing senescence-exacerbating pathways like IL16 and GH. By enhancing signal integration in MAIT/NKT cells and reducing CD8^+^ TEM’s reliance on external inputs, HUC-MSCs transform the fragmented immune dialogue of aging into a coordinated network. The combined amplification of protective crosstalk and silencing of inflammatory noise restores systemic immune competence, demonstrating a precision therapeutic approach to counteract immunosenescence in age-related frailty.

### 3.7 Immune Remodeling Correlates with Clinical Improvement Following HUC-MSCs Therapy

We performed Spearman correlation analyses to examine whether the observed immune remodeling was associated with the clinical response to HUC-MSC therapy. Patient-level changes in immune cell subset proportions were correlated with changes in clinical metrics at matched time points (1- and 2-months post-infusion; **Supplementary Figure 4**). The full correlation matrix is provided in **Supplementary Table 3**. At 1-month post-treatment, expansion of regulatory T cells (Tregs) positively correlated with improvement in the FTSST (ρ = +0.66, p = 0.019, n = 12), suggesting that greater Treg increases were associated with greater gains in lower-limb function. Furthermore, upregulation of TUBB1in megakaryocytes correlated with improved 4MWT performance (ρ = +0.62, p = 0.043, n = 11), aligning with the megakaryocyte maturation phenotype described in Section 3.3. By 2 months post-treatment, clinical improvement was associated with reductions in several B cell and innate immune populations. Decreases in memory B cell (ρ = −0.72, p = 0.013, n = 11) and naïve B cell proportions (ρ = −0.61, p = 0.047, n = 11) correlated with greater improvement in grip strength, consistent with the attenuation of B cell hyperactivation noted in Section 3.2. Reductions in megakaryocyte (ρ = −0.66, p = 0.029, n=11) and monocyte proportions (ρ = −0.65, p = 0.032, n=11) were associated with better 4MWT performance. Notably, the positive correlation between Treg expansion and FTSST improvement persisted at this time point (ρ = +0.65, p = 0.031, n = 11). The directionality of these associations, which is consistent with the proposed biological narrative, is summarized in heatmap visualizations (**Supplementary Figure 4**). Among 204 exploratory pairwise correlations tested, 10 were nominally significant (p < 0.05). This number approximates the rate expected by chance (~10.2 at α = 0.05), and none of the correlations survived Benjamini–Hochberg correction for multiple testing (**Supplementary Table 3**). Therefore, these results should be interpreted as hypothesis-generating, providing preliminary evidence for a biologically coherent link between specific immune changes and clinical improvement, rather than as confirmatory findings.

## 4 Discussion

The clinical assessments in this exploratory study suggest that HUC-MSC treatment was associated with trends of improvement in frailty status and physical function—including mobility, strength, and overall performance—as measured by the Fried scale, SPPB, TUG, 4MWT, grip strength, and FTSST (**Supplementary Table 1**). These changes were observable as early as 1 month and appeared maintained at 2 months, whereas the control group did not show a similar trajectory. Although these clinical findings are preliminary and must be interpreted cautiously due to the limited sample size, they align with previous reports indicating that MSC therapy may confer functional benefits in older adults with frailty (19–21). This correlation motivates the investigation of immune alterations at the single−cell level to explore whether the observed clinical changes are linked to the immunomodulatory properties of MSCs. Our study delineates a multi-faceted immunomodulatory framework through which HUC-MSCs therapy counteracts aging-related frailty, a condition hallmarked by inflammaging and immune dysregulation (32, 33). By integrating single-cell transcriptomics and ligand-receptor network analysis, we demonstrate that HUC-MSCs orchestrate dynamic, cell type-specific reprogramming of both innate and adaptive immunity, culminating in restored immune equilibrium. The transient increase in peripheral MAIT cells at 1-week post-treatment (*p* = 0.049; **Figure 4c**) suggests an early immunomodulatory shift. Based on the established biology of MAIT cells, this expansion may potentially support a rapid response phase, which could involve enhanced immune surveillance; however, their specific roles in pathogen control or senescent cell clearance in this context remain to be directly investigated. Subsequently, the resolution of this expansion may align with a transition towards tissue homing and inflammation resolution, processes often mediated by factors such as *IL-10* and *TGF-β* (30). Concurrently, B cells demonstrated a unique pattern of activation and modulation, characterized by the downregulation of antigen presentation genes (*HLA-DRB5*, *HLA-DQA2*, *HLA-C*, and *NKG7*) and the upregulation of activation markers (*HLA-DRB1*, *RPS26*, *JUND*, *IGLC2*, and *EZR*) (**Figure 5e**). This activation-with-restraint phenotype indicates that HUC-MSCs recalibrate B cell function to enhance T cell priming while mitigating age-related hyperactivation, potentially reversing the anergy typically observed in elderly B cell responses (34). NK cells further exemplified the dual regulatory role of HUC-MSCs: genes associated with cytotoxicity (*GNLY*) were upregulated, thereby reinforcing innate immune surveillance, whereas pro-inflammatory mediators (*DUSP2*, *HSPA1A*) were downregulated (**Figure 5c**). This balanced response—characterized by enhanced effector function coupled with reduced inflammation—corresponds with the role of MSCs in modulating immune homeostasis without compromising host defense (35). Similarly, NKT cells exhibited a shift in stress-response signaling, with an upregulation of *PPDPF* and a downregulation of *RPS11*, *FAM118A*, and *ITGB2* (**Supplementary Figure 2b**), promoting tolerance over excessive activation. The central mechanism for system-wide immune coordination involved the HUC-MSCs-mediated rewiring of intercellular networks. The EFNA2-EPHA2 axis dominated immune crosstalk (**Figure 6g**), repurposing CD4^+^ TEM and MAIT/NKT cells as proactive signaling hubs while silencing maladaptive pathways (*e.g*., *IL16*). This reconfiguration mirrors youthful immune dynamics, where adaptive-innate crosstalk ensures precise response coordination (36). This reorganization of cell-cell communication represents a core mechanism through which HUC-MSCs therapy may resolve chronic inflammation, offering a coordinated strategy to counteract inflammaging.

Our integrative correlation analyses provide initial quantitative support for the link between HUC-MSC-induced immune remodeling and functional recovery. The reductions in B cell subsets at 2 months correlated with improved grip strength (Memory B: ρ = −0.72; Naïve B: ρ = −0.61), aligning with the hypothesis that attenuating chronic B cell hyperactivation contributes to improved muscular function—a mechanism consistent with established inflammaging models of frailty. The sustained positive association between Treg expansion and improved FTSST at both 1 and 2 months (ρ = +0.66 and +0.65, respectively) further supports the role of enhanced immune regulation in resolving the chronic inflammation underlying sarcopenia. Additionally, reductions in megakaryocyte and monocyte proportions at 2 months, alongside earlier TUBB1 upregulation in megakaryocytes, were associated with improved gait speed, suggesting that innate-cell remodeling also contributes to physical recovery.

Crucially, these findings must be interpreted within the constraints of our pilot study. With a cohort of n=12, statistical power is limited, and no association survived multiple-testing correction. Therefore, the directional coherence​ between specific immune changes and clinical improvement—rather than any single p-value—provides the strongest evidence that the observed transcriptional reprogramming is biologically linked to benefit. These results are hypothesis-generating and warrant validation in larger cohorts. Future studies employing pre-specified immune endpoints and longitudinal mixed-effects models are needed to formally test the predictive value of these immune signatures for clinical response.

## 5 Conclusion

Our findings suggest that HUC-MSCs may modulate multiple aspects of aged immunity, influencing both cellular states and intercellular communication networks. This pilot study provides evidence of short-term safety and immunomodulatory activity. Larger trials with extended follow-up are needed to validate the durability of these effects and to optimize dosing. Future mechanistic work should focus on deciphering how MSC-secreted factors (e.g., exosomes, metabolites) spatially regulate immune cell trafficking and tissue repair to advance clinical translation. By promoting a more balanced immune state across multiple compartments, HUC-MSC therapy represents a promising, biology-driven approach for managing aging-related frailty.

## 6 Limitations and future directions

The findings of this study should be interpreted within its exploratory framework and inherent constraints. First, the primary limitation is the modest and unbalanced sample size (n=12), which, despite offering valuable longitudinal insights, may increase the risk of confounding effects from individual biological variability. The final cohort size was constrained by operational challenges and budget limitations during the trial period, which coincided with the COVID-19 pandemic. Consequently, the single-cell data herein function as a discovery cohort to map potential immunomodulatory mechanisms; future validation in larger, independent cohorts is essential.

Moreover, the mechanistic insights drawn—specifically regarding MAIT cell tissue homing and the involvement of the EPHA/NRG signaling pathways—remain primarily correlative.​ While these associations are strongly supported by our bioinformatic analysis and prior literature, they originate from observational clinical data and lack direct functional validation. This study’s design, as an initial exploratory trial, prioritized defining the comprehensive immune landscape and was not powered to perform concomitant functional perturbations. Therefore, these pathways should be considered compelling, hypothesis-generating findings rather than definitively established mechanisms.

To advance from correlation to causation, future work must include targeted functional studies. *In vitro* co-culture assays (e.g., utilizing receptor blockade or neutralizing antibodies) and *in vivo* perturbation models are needed to confirm the causal roles of these pathways. Additionally, the application of spatial transcriptomics could provide critical insights into the spatial dynamics of immune cell trafficking and interactions within tissues post-MSC therapy. Finally, while promising short-term outcomes were observed, definitive conclusions regarding the long-term durability of the immune restoration and the optimal therapeutic dosing regimen require larger-scale, long-term clinical studies.

## Data Availability

The raw sequence data reported in this paper have been deposited in the Genome Sequence Archive (37) in National Genomics Data Center (38), China National Center for Bioinformation / Beijing Institute of Genomics, Chinese Academy of Sciences (GSA-Human: HRA010756) that are publicly accessible at https://ngdc.cncb.ac.cn/gsa-human/.

## Supplementary material

Supplementary Figure 1 Batch effect correction using Harmony integration. UMAP visualization of all 339,437 PBMCs colored by sample identity (42 samples) before (left) and after (right) Harmony batch correction.

Supplementary Figure 2 HUC-MSC therapy induces cell-type-specific transcriptional reprogramming. **(a)** Bar plot depicting the number of differentially expressed genes (DEGs) identified in each major immune cell type at the indicated post-treatment time points. **(b)** Visualization of gene expression dynamics shows that *KLRB1*and *DUSP1* were commonly upregulated in MAIT and NKT cells, while *FOS* and *JUN* were commonly downregulated in CD4^+^ naïve T cells, CD8^+^ TEM, and regulatory T cells across all time points. **(c, d)** Dot plots of significantly enriched Gene Ontology (GO) terms. c. In MAIT cells, enriched terms were associated with “response to cytokine stimulation” and “regulation of T cell activation”. d. In naïve B cells, enriched terms included “antigen processing and presentation” and “MHC class II complex assembly”, indicating a shift toward adaptive immune priming functions. Dot size represents the significance of enrichment (-log_10_(p-value)).

Supplementary Figure 3 HUC-MSC therapy reprograms intercellular communication, including NRG-mediated signaling, in aging frailty. **(a)** Overview of the global cell-cell communication networks. The HUC-MSC-treated group exhibited strengthened interactions between CD8^+^ TEM and regulatory T cells (Tregs), whereas signaling initiated by CD4^+^ naïve T cells was attenuated. **(b)** Temporal dynamics of T cell–B cell interactions visualized by heatmap. Communication between CD4^+^ TEM and memory B cells was notably enhanced at 2 months, with CD8^+^ TEM serving as the dominant signal-receiving cluster. **(c)** Signaling architecture of the NRG pathway. NKT cells functioned as the primary signal senders, while CD8^+^ TEM and MAIT cells acted as the major receivers in the HUC-MSC group. Edge thickness corresponds to interaction probability (strength).

Supplementary Figure 4 Immune-clinical correlation heatmaps at 1 and 2 months post-HUC-MSC treatment. Spearman correlation analysis between patient-level changes (Δ) in immune cell subset proportions and changes in clinical frailty/physical performance metrics. **(a)** 1-month and **(b)** 2-month time points. Positive Δ values indicate an increase in cell proportion or an improvement in the clinical metric. Color scale: red, positive correlation (ρ); blue, negative correlation. Correlation coefficients (Spearman’s ρ) are overlaid.
