# Correction: Immunomodulatory effects of HUC-MSCs therapy: enhancing adaptive and innate immune responses in aging frailty

**DOI:** 10.3389/fimmu.2026.1938134

**Published:** 2026-07-23

**Authors:** Ce Huang, Yingqian Zhu, Xue Gong, Shengyu Feng, Guangying Huo, Hailiang Liu, Hua Jiang, Zhongmin Liu

**Affiliations:** 1 Institute for Regenerative Medicine, State Key Laboratory of Cardiology and Medical Innovation Center, Shanghai East Hospital, School of Medicine, Tongji University, Shanghai, China; 2 Institutes of Biomedical Sciences, Shanghai Institute of Infectious Disease and Biosecurity, Fudan University, Shanghai, China; 3 Department of Geriatrics, Shanghai East Hospital, Tongji University School of Medicine, Shanghai, China; 4 Department of General Medicine, Shanghai East Hospital, Tongji University School of Medicine, Shanghai, China; 5 Key Laboratory of Xinjiang Phytomedicine Resource and Utilization of Ministry of Education, College of Life Sciences, Shihezi University, Shihezi, China; 6 Institute of Advanced Biotechnology and School of Medicine, Southern University of Science and Technology, Shenzhen, China

**Keywords:** human umbilical cord-derived mesenchymal stem cells (HUC-MSCs), aging frailty, single-cell RNA sequencing, mucosal-associated invariant T (MAIT) cells, cell-cell communication, immunosenescence

An incorrect **Funding** statement was provided. The correct funder is Shanghai “Rising Stars of Medical Talents” Youth Development Program. The correct **Funding** statement reads:

“The author(s) declared that financial support was received for this work and/or its publication. This work was supported by the Peak Disciplines (Type IV) of Institutions of Higher Learning in Shanghai (Stem Cells and Translation), the National Natural Science Foundation of China (82271593), the Fundamental Research Funds for the Central Universities (22120240281), the Natural Science Foundation of Shanghai under the Shanghai Action Plan for Science, Technology and Innovation (24ZR1469200), the Tongji University Medicine-X Interdisciplinary Research Initiative (2025-0554-YB-18; 2026-0554-YB-16), the National Key Research and Development Program of China (2022YFC3601504), and the Shanghai “Rising Stars of Medical Talents” Youth Development Program - Youth Medical Talents - General Practitioner Program (SHWSRS(2025)_071).”

Incorrect correspondence emails were provided for authors “Zhongmin Liu and Hailiang Liu. The correct correspondence list should read:

Zhongmin Liu, liu.zhongmin@tongji.edu.cn; Hua Jiang, huajiang2013@tongji.edu.cn; Hailiang Liu, hailiang_1111@tongji.edu.cn”

The original version of this article has been updated.

