# Supplementary material for: Immunomodulatory effects of HUC-MSCs therapy: enhancing adaptive and innate immune responses in aging frailty
Source: Front Immunol. 2026 Jul 13;17:1796161. doi: 10.3389/fimmu.2026.1796161 (PMC13444523; doi:10.3389/fimmu.2026.1796161)
Supplement: Supplementary file 1 [file SupplementaryFile1.docx]

**Supplementary Figure 1.** **Batch effect correction using Harmony integration.** UMAP visualization of all 339,437 PBMCs colored by sample identity (42 samples) before (left) and after (right) Harmony batch correction.

**Supplementary Figure 2. HUC-MSC therapy induces cell-type-specific transcriptional reprogramming.**

**a**, Bar plot depicting the number of differentially expressed genes (DEGs) identified in each major immune cell type at the indicated post-treatment time points. **b**, Visualization of gene expression dynamics shows that *KLRB1*and *DUSP1* were commonly upregulated in MAIT and NKT cells, while *FOS* and *JUN* were commonly downregulated in CD4^+^ naïve T cells, CD8^+^ TEM, and regulatory T cells across all time points. **c**, **d**, Dot plots of significantly enriched Gene Ontology (GO) terms. c, In MAIT cells, enriched terms were associated with "response to cytokine stimulation" and "regulation of T cell activation". d, In naïve B cells, enriched terms included "antigen processing and presentation" and "MHC class II complex assembly", indicating a shift toward adaptive immune priming functions. Dot size represents the significance of enrichment (-log₁₀(p-value)).

**Supplementary Figure 3. HUC-MSC therapy reprograms intercellular communication, including NRG-mediated signaling, in aging frailty.**

**a,** Overview of the global cell-cell communication networks. The HUC-MSC-treated group exhibited strengthened interactions between CD8^+^ TEM and regulatory T cells (Tregs), whereas signaling initiated by CD4^+^ naïve T cells was attenuated. **b,** Temporal dynamics of T cell–B cell interactions visualized by heatmap. Communication between CD4^+^ TEM and memory B cells was notably enhanced at 2 months, with CD8^+^ TEM serving as the dominant signal-receiving cluster. **c,** Signaling architecture of the NRG pathway. NKT cells functioned as the primary signal senders, while CD8^+^ TEM and MAIT cells acted as the major receivers in the HUC-MSC group. Edge thickness corresponds to interaction probability (strength).

**Supplementary Figure 4. Immune-clinical correlation heatmaps at 1 and 2 months post-HUC-MSC treatment.**

Spearman correlation analysis between patient-level changes (Δ) in immune cell subset proportions and changes in clinical frailty/physical performance metrics. (a) 1-month and (b) 2-month time points. Positive Δ values indicate an increase in cell proportion or an improvement in the clinical metric. Color scale: red, positive correlation (ρ); blue, negative correlation. Correlation coefficients (Spearman's ρ) are overlaid.
